# Supplementary material for: Empirical methods for controlling false positives and estimating confidence in ChIP-Seq peaks
Source: BMC Bioinformatics. 2008 Dec 5;9:523. doi: 10.1186/1471-2105-9-523 (PMC2628906; doi:10.1186/1471-2105-9-523)
Supplement: Additional File 1 — A variety of html documents from the USeq web site detailing the available applications, their best usage, output file type descriptions, command line menus, etc. [file 1471-2105-9-523-S1.zip › USeqUserGuides/JavaDoc.zip!/JavaDoc/trans/qc/CelFileQualityControl.html]

CelFileQualityControl


|  |  |  |
| --- | --- | --- |
| **Overview** \| **Package** \| **Class** \| **Use** \| **Tree** \| **Deprecated** \| **Index** \| **Help** | |  |
| PREV CLASS   **NEXT CLASS** | **FRAMES**    **NO FRAMES**     **All Classes** |  |
| SUMMARY: NESTED \| FIELD \| CONSTR \| METHOD | DETAIL: FIELD \| CONSTR \| METHOD |  |


---


## trans.qc Class CelFileQualityControl

```
java.lang.Object
  trans.qc.CelFileQualityControl
```

---

``` public class CelFileQualityControl extends java.lang.Object ```

Provides automated qc checking for cel files, flags those of poor quality
based on a variety of statistics.
For every cel file, the median intensity is calculated, then a median determined for all the cel files' medians,
then a scaler is calculated for each cel file required to get it to the overall median. This is the cel file specific
median scalar and indicates the degree of brighness relative to the other chips. After this calculation the intensities
on all the chips are median scaled to the targetMedian defined in the application, ie 100. Lastly, all the stats are
calculated on these scaled values.
For the dim bright no synth calculations, these are made using the particular chip intensities, not any global values.

**Author:**
:   David Nix

---

**Constructor Summary**

`CelFileQualityControl()`


| **Method Summary** | |
| --- | --- |
| `void` | `calculateMedianStatFlagValues()`             Calculates the median value for each stat flag |
| `void` | `checkForFlippedControls()`             Runs through groups of cel files, calculates the median no Synth, Dim, and Bright intensities, checks to see if they are inverted, for some reason they sometimes are! Note these medians are calculated on the raw, pre scaled chip intensities. |
| `void` | `checkSingleCelFileStats()`             Runs through groups of cel files, calling statSingleCelFile() on each and prints the results. |
| `void` | `cluster(int[][] pmCoordinates)`             Clusters cel files based on pm intensities by group. |
| `void` | `clusterCelFiles(int[][] pmCoordinates)`             Runs through groups of cel files, calling statSingleCelFile() on each and prints the results. |
| `void` | `createCelFileStatsArray()`             Creates a cel file stats array, then saves and nulls the float arrays. |
| `java.io.File[]` | `fetchCelFileStatsFiles()` |
| `java.lang.String` | `fetchQCParams()` |
| `void` | `instantiateStatFlags()`             Makes the Stat Flags, add new stats here. |
| `static void` | `main(java.lang.String[] args)` |
| `void` | `makeCharts()`             Writes charts to the save directory and if so indicated displays them. |
| `void` | `printCelFileStats()`             Runs through groups of cel files, calling statSingleCelFile() on each and prints the results. |
| `static void` | `printDocs()` |
| `void` | `printStatFlags()`             Prints name, median min max for each stat flag |
| `void` | `processArgs(java.lang.String[] args)`             This method will process each argument and assign any new varibles |
| `java.lang.StringBuffer` | `statSingleCelFile(CelFileStats cs)`             Checks a CelStats object for deviations from the min max settings. |
| `void` | `testCelFiles()`             Main app controler. |
| `void` | `writePNGs(boolean restrictToOutlier)`             Writes PNG files for CelFileStats. |

**Methods inherited from class java.lang.Object**

`equals, getClass, hashCode, notify, notifyAll, toString, wait, wait, wait`

**Constructor Detail**

### CelFileQualityControl

```
public CelFileQualityControl()
```


**Method Detail**

### testCelFiles

```
public void testCelFiles()
```

:   Main app controler.

---


### cluster

```
public void cluster(int[][] pmCoordinates)
```

:   Clusters cel files based on pm intensities by group.

---


### makeCharts

```
public void makeCharts()
```

:   Writes charts to the save directory and if so indicated displays them.

---


### checkForFlippedControls

```
public void checkForFlippedControls()
```

:   Runs through groups of cel files, calculates the median no Synth, Dim, and Bright intensities,
    checks to see if they are inverted, for some reason they sometimes are! Note these medians
    are calculated on the raw, pre scaled chip intensities.

---


### printCelFileStats

```
public void printCelFileStats()
```

:   Runs through groups of cel files, calling statSingleCelFile() on each
    and prints the results.

---


### clusterCelFiles

```
public void clusterCelFiles(int[][] pmCoordinates)
```

:   Runs through groups of cel files, calling statSingleCelFile() on each
    and prints the results.

---


### checkSingleCelFileStats

```
public void checkSingleCelFileStats()
```

:   Runs through groups of cel files, calling statSingleCelFile() on each
    and prints the results.

---


### fetchCelFileStatsFiles

```
public java.io.File[] fetchCelFileStatsFiles()
```

---


### writePNGs

```
public void writePNGs(boolean restrictToOutlier)
```

:   Writes PNG files for CelFileStats.

---


### printStatFlags

```
public void printStatFlags()
```

:   Prints name, median min max for each stat flag

---


### calculateMedianStatFlagValues

```
public void calculateMedianStatFlagValues()
```

:   Calculates the median value for each stat flag

---


### instantiateStatFlags

```
public void instantiateStatFlags()
```

:   Makes the Stat Flags, add new stats here. Set

---


### createCelFileStatsArray

```
public void createCelFileStatsArray()
```

:   Creates a cel file stats array, then saves and nulls the float arrays.

---


### statSingleCelFile

```
public java.lang.StringBuffer statSingleCelFile(CelFileStats cs)
```

:   Checks a CelStats object for deviations from the min max settings.
    appends nothing if no errors.

---


### main

```
public static void main(java.lang.String[] args)
```

---


### processArgs

```
public void processArgs(java.lang.String[] args)
```

:   This method will process each argument and assign any new varibles

---


### fetchQCParams

```
public java.lang.String fetchQCParams()
```

---


### printDocs

```
public static void printDocs()
```


---


|  |  |  |
| --- | --- | --- |
| **Overview** \| **Package** \| **Class** \| **Use** \| **Tree** \| **Deprecated** \| **Index** \| **Help** | |  |
| PREV CLASS   **NEXT CLASS** | **FRAMES**    **NO FRAMES**     **All Classes** |  |
| SUMMARY: NESTED \| FIELD \| CONSTR \| METHOD | DETAIL: FIELD \| CONSTR \| METHOD |  |


---
